# Increased heartbeat-evoked potential during REM sleep in nightmare disorder

**DOI:** 10.1016/j.nicl.2019.101701

**Published:** 2019-01-29

**Authors:** Lampros Perogamvros, Hyeong-Dong Park, Laurence Bayer, Aurore A. Perrault, Olaf Blanke, Sophie Schwartz

**Affiliations:** a Center for Sleep Medicine, Division of Pulmonology, Department of Medical Specialties, Geneva University Hospitals, Geneva, Switzerland; b Department of Neuroscience, Faculty of Medicine, University of Geneva, Geneva, Switzerland; c Swiss Center for Affective Sciences, University of Geneva, Geneva, Switzerland; d Laboratory of Cognitive Neuroscience, Center for Neuroprosthetics and Brain Mind Institute, Ecole Polytechnique Fédérale de Lausanne, Geneva, Switzerland; e Department of Neurology, University of Geneva, Switzerland

**Keywords:** Nightmares, REM sleep, Heartbeat-evoked potential, EEG, Emotional arousal

## Abstract

Nightmares are characterized by the experience of strong negative emotions occurring mainly during REM sleep. Some people suffer from nightmare disorder, which is defined by the repeated occurrence of nightmares and by significant distress in wakefulness. Yet, whether frequent nightmares relate to a general increase in emotional reactivity or arousal during sleep remains unclear. To address this question, we recorded heartbeat-evoked potentials (HEPs) during wakefulness, NREM and REM sleep in patients with nightmare disorder and healthy participants. The HEP represents a cortical (EEG) response to the heartbeat and indexes brain-body interactions, such as interoceptive processing and intrinsic levels of arousal. HEP amplitude is typically increased during states of high emotional arousal and motivation, and is decreased in depression. Here we compared the amplitude of HEPs between nightmare patients and healthy controls separately during AWAKE, NREM, REM periods, and found higher HEP amplitude in nightmare patients compared to healthy controls over a cluster of frontal regions only during REM sleep. This effect was not paralleled by any group difference in cardiac control measures (e.g. heart rate variability, interbeat interval). These findings corroborate the notion that nightmares are essentially a REM pathology and suggest that increased emotional arousal during REM sleep, as measured by HEP, is a physiological condition responsible for frequent nightmares. This result also supports that HEP may be used as a biomarker of increased emotional and sensory processing during REM sleep in these patients.

## Highlights

• We used heartbeat-evoked EEG potential (HEP) as an index of emotional arousal.
• Nightmare patients had higher HEP amplitude during REM sleep compared to controls.
• This increase specifically concerned a cluster of frontal electrodes.
• Higher HEP indicates elevated emotional arousal during REM sleep in this disorder.

## 1 Introduction

Nightmare disorder is a parasomnia characterized by extremely dysphoric dreams, usually occurring during REM sleep (American Academy of Sleep Medicine, 2014; American Psychiatric Association, 2013). Nightmares may involve images, feelings or thoughts of physical aggression, interpersonal conflict, failure/helplessness, and emotions like fear, anxiety, anger, sadness, and disgust (Robert and Zadra, 2014). The prevalence of nightmare disorder at a clinically significant frequency (>1 episode per week) varies from 1% up to 7% of individuals (Munezawa et al., 2011; Sandman et al., 2013). Nightmares may be idiopathic (without clinical signs of psychopathology) or associated with a broad range of other disorders including post-traumatic stress disorder (PTSD), substance abuse, depression, stress and anxiety, borderline personality disorder, schizophrenia, and other psychiatric illnesses.

The pathophysiology of nightmare disorder remains largely unknown. It has been proposed that nightmare disorder involves a dysfunction of a network that encompasses limbic, paralimbic and prefrontal regions (e.g. amygdala, medial prefrontal cortex, hippocampus, anterior cingulate cortex, insula), which may also explain why patients present altered emotion regulation in response to stressors that are temporary (e.g., daily concerns) or persistent (e.g., trauma) (Nielsen and Levin, 2007). A genetic component has been documented (Coolidge et al., 2010; Hublin et al., 1999) although the functional role of this contribution is not well understood.

As experiences occurring during sleep, nightmares might reflect intensified emotional arousal and heightened emotional reactivity during dreaming. Fear is the most frequently reported emotion in nightmares, while physical aggression is the most reported theme (Robert and Zadra, 2014). Physiologically, heightened arousal during the sleep of nightmare recallers is suggested by increased leg movements (Germain and Nielsen, 2003), increased high alpha power (Simor et al., 2013) and more frequent awakenings (Simor et al., 2012). One study also reported that patients with nightmare disorder also have a high sympathetic drive during REM sleep (Nielsen et al., 2010). More specifically, during post-deprivation recovery sleep, nightmare subjects were found to show higher than normal low-frequency spectral power in the ECG, which reflects sympathetic influences on the heart, and low high-frequency spectral power, which reflects respiration-driven vagal modulation of the heart (Nielsen et al., 2010). These changes were most prominent in REM sleep. In terms of waking personality traits, nightmare sufferers have been found to be more open, sensitive, and affected by experiences, including being more vulnerable to stress and trauma (Hartmann, 1989). These patients score higher on both state (Schredl, 2003) and trait (Spoormaker et al., 2006) anxiety, neuroticism (Blagrove et al., 2004), novelty seeking and anticipatory worry (Perogamvros et al., 2015), and show heightened physical and emotional reactivity (Kales et al., 1980; Levin, 1994) and maladaptive coping (Köthe and Pietrowsky, 2001). A breakdown of emotion regulation processes such as fear extinction has been suggested to be happening during nightmares, resulting in emotional dysfunction, as found in depression and PTSD (Nielsen and Levin, 2007). Because nightmare disorder is described as primarily affecting fear-related processes (American Academy of Sleep Medicine, 2014; American Psychiatric Association, 2013), it may reflect a pathological breakdown of the normal functioning of fear expression, memory and regulation during sleep and dreaming (Nielsen and Carr, 2017).

Heightened sensory processing sensitivity has been recently described as an appropriate trait to describe nightmare sufferers (Carr and Nielsen, 2017). Amplified sensory processing sensitivity involves a deeper cognitive processing of both external and internal information that is driven by higher emotional reactivity (Aron et al., 2012). A plethora of theories and experimental observations supports that the perception of bodily signals is a key component of emotional experience (Damasio, 1999; James, 1884). Hence, measuring the cortical representation of bodily responses elicited by emotional events may serve as a reliable neural marker of affective states and emotional arousal (Mussgay et al., 1999; Schandry, 1981). In this context, the heartbeat evoked potential (HEP), occurring about 200–600 ms after the R-peak of the ECG waveform (Schandry et al., 1986), has emerged as a useful tool to assess interoceptive processing (Ishii and Canuet, 2012; Lechinger et al., 2015; Park et al., 2016; Park et al., 2014). Critically, HEP amplitude is increased in states of high emotional arousal (Luft and Bhattacharya, 2015), heightened motivation (Weitkunat and Schandry, 1990), and stress (Schulz et al., 2013). Conversely, HEP amplitude is reduced in depression (Terhaar et al., 2012), consistent with decreased bodily awareness, alexithymia (i.e. inability to experience emotions), and blunted emotional reactivity found in depressed patients (Bylsma et al., 2008; Imbault and Kuperman, 2018).

Based on the literature reviewed above, we hypothesized that patients with nightmare disorder may show increased emotional arousal predominantly during REM sleep. We therefore investigated HEPs during wakefulness, NREM and REM sleep in patients with nightmare disorder and healthy controls. We expected that HEP amplitude would be higher (more positive) in nightmare disorder during REM sleep, reflecting increased emotional arousal during this sleep stage. Because highly negative emotions engage the amygdala, insula, and anterior cingulate cortex, we also predicted that HEP increases, as measured by scalp EEG, would predominate over frontal electrodes around 200 to 600 ms post R-peak time window. Finally, we examined whether HEP measures may also correlate with depression level, as previously reported.

## 2 Methods

### 2.1 Participants

Eleven carefully selected patients with nightmare disorder were included (8 females, age 34 ± 9.3 years (mean ± s.d.)). The patients sought consultation on their own or were referred by medical doctors of the Geneva area because of intense dreaming with negative emotional content. During the first consultation, diagnosis of nightmare disorder was done by a sleep specialist according to the International Classification of Sleep Disorders (ICSD-3) diagnostic and coding manual (American Academy of Sleep Medicine, 2014). In addition, a neuropsychiatric evaluation was performed to assess possible comorbidities, such as depression (measured by Beck Depression Inventory/ BDI-II, (Beck et al., 1996)), psychosis or anxiety disorder. We excluded any patient with symptoms of obstructive sleep apnea syndrome, restless legs syndrome, or using medications that would be likely to produce nightmares (e.g. hypnotics, β-blockers, amphetamines, antimicrobial agents); any patient with moderate or severe depression (Beck Depression Inventory Score > 17), generalized anxiety disorder, PTSD, known psychotic disorder; and any patient with a neurological disease.

Eleven age and sex-matched healthy good sleepers controls were also included (8 females, age 33 ± 9.2 years (mean ± s.d.)). They all had no history of neurological, psychiatric or sleep disorder, including absence of nightmare disorder. All controls had <1 nightmare at home during the past month.

Signed informed consent was obtained from all participants before the experiment, and ethical approval for the study was obtained from the Ethical Committee of the Geneva University Hospitals.

### 2.2 Polysomnography

Polysomnography (DeltaMed Coherence PSG, Natus, France) was recorded using 20 EEG electrodes according to the international 10–20 system (Fp1, Fp2, F3, F4, F7, F8, C3, C4, P3, P4, O1, O2, T3, T4, T5, T6, Fz, Cz, Pz, Fpz (Ref)). Right and left electrooculogram (EOG), chin electromyogram (EMG) and electrocardiogram (ECG) were recorded using conventional bipolar recording leads. To control for the presence of apnea and hypopnea, nasal and oral airflows were recorded with a pressure transducer (Protech2, Minneapolis, MN, USA) and thoracic and abdominal respiratory movements were acquired with strain gauges. Oxygen saturation (SaO2) was continuously measured with a finger oximeter. Right and left anterior tibialis muscle EMG activity was recorded using bipolar surface electrodes to monitor lower limb motor activity. EEG and EMG signals were sampled at 512 Hz. Sleep was scored according to the AASM Manual for the Scoring of Sleep and Associated Events (Berry et al., 2012).

### 2.3 Assessment of negative affect

In order to assess the propensity of nightmare sufferers to experience negative affect, we used the Beck Depression Inventory ((BDI-II, (Beck et al., 1996)). Based on previous literature (Terhaar et al., 2012), we made the a priori directional hypothesis that, as an index of emotional reactivity, HEP amplitude in emotion-related regions (e.g. frontal, see Introduction) during REM sleep would negatively correlate with individual BDI scores in nightmare patients. Note that for the control group, we included the BDI scores for 10 participants, as one participant did not complete this questionnaire.

### 2.4 EEG analysis

Preprocessing and averaging were conducted using Fieldtrip toolbox (Oostenveld et al., 2011). Continuous EEG and ECG data were down-sampled to 256 Hz, offline filtered between 1 and 40 Hz. EEG data were re-referenced to a common average reference. Independent component analysis (ICA) was conducted on the continuous EEG signals and stereotypical independent components reflecting eye movements and eye blinks were removed based on the visual inspection of all the independent components (Delorme and Makeig, 2004).

For HEP analysis, we first selected all the continuous time windows representing at least 5 min spent in one sleep stage or wakefulness, and then concatenated them separately in AWAKE, REM and NREM conditions. Heartbeat evoked potentials (HEP) were computed on EEG signals locked to the R-peak of the ECG, separately for each condition (Park et al., 2014). We detected R-peaks on ECG by correlating the ECG signal with a template QRS complex defined on a subject-by-subject basis, and identified local maxima within episodes of correlation larger than 0.7. Epochs (-300 to 700 ms regarding the R-peak onset) showing excessive noise (i.e., >3 SD) were excluded from further analysis. After artifact correction, 6431/2819, 11601/10896, and 4084/3570 epochs were averaged to compute HEP, respectively for AWAKE, NREM (merged N2 and N3 stages), and REM periods in nightmare/control groups.

**Fig. 1:**
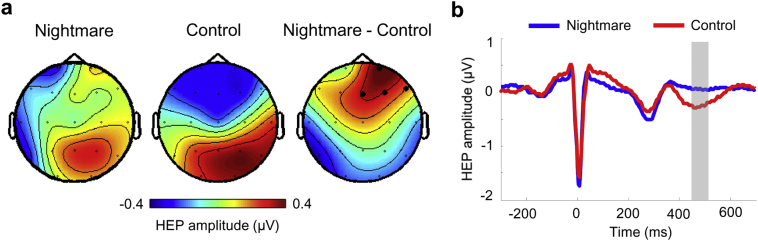
More positive HEP amplitude during REM sleep in nightmare disorder. a. Topographical map of the HEP amplitude during the REM sleep in nightmare (left), control groups (middle), and their differences (nightmare minus control; right) in the 449–504 ms time window in which a significant difference was observed. b. Time course of HEP at the electrodes indicated by a black dot in a, which showed significant HEP differences. The shaded area highlights the time window in which a significant difference was observed (cluster level *p* = .032, *n* = 11).

In addition, HEP is known to be heavily contaminated by ECG artifacts, as the ECG can be recorded even at scalp electrodes overlying cortical regions (see Fig. 1). To check the possibility that possible differential HEP amplitude between nightmare and control groups did not result from such ECG-based EEG difference, we further analyzed whether any HEP difference is accompanied by ECG difference in the same time window (Park et al., 2016, 2014), without applying ICA to attenuate ECG artifact.

Furthermore, we also checked whether possible HEP differences were associated with differences in interbeat interval or heart rate variability. Mean interbeat interval was computed by averaging intervals between two consecutive ECG R-peaks for each condition considering the continuous time windows in which HEP were assessed. Similarly, heart rate variability (i.e., SDNN: standard deviation of normal-to-normal interbeat intervals) was obtained by computing the standard deviation of the interbeat intervals for each condition (Quintana et al., 2016).

### 2.5 Statistical analysis

HEPs difference between nightmare and control groups was tested using the cluster-based permutation *t*-test (Maris and Oostenveld, 2007) as implemented in the Fieldtrip toolbox (Oostenveld et al., 2011). Individual samples whose t-value exceeded a threshold (*p* < .05, two-tailed) were clustered based on temporal and spatial adjacency. Each cluster defined in time and space by this procedure was assigned cluster-level statistics, corresponding to the sum of the t-values of the samples belonging to that cluster. To define neighboring electrodes, a triangulation algorithm was used. This method generates triangles between nearby electrodes, and is not affected by distance between electrodes. A minimum of two significant electrodes was considered as a cluster. Type-I error rate was controlled by evaluating the maximum cluster-level statistics under the null hypothesis: condition labels were randomly shuffled 1000 times to estimate the distribution of maximal cluster-level statistics obtained by chance. The two-tailed Monte-Carlo *p*-value corresponded to the proportion of the elements in the distribution of shuffled maximal cluster-level statistics that exceeded the observed maximum or minimum original cluster-level test statistics. Because this method uses maxima, it intrinsically corrects for multiple comparisons in time and space. This procedure was applied at the electrode level in the time window from 200 to 600 ms after the R-peaks.

## 3 Results

### 3.1 Clinical characteristics

In the nightmare group, the frequency of nightmares at home was 3.9 ± 2.1 episodes per week (mean ± SD, range: 1–7 episodes per week). Three subjects had a well-remembered nightmare in the laboratory (without associated awakening), as reported in a morning dream diary, whereas no nightmares were reported in controls. The nightmare and control groups significantly differed for mood scores (BDI mean ± SD: nightmares = 8 ± 4.5; controls = 1.4 ± 1.9; *p* = .002, Mann-Whitney *U* test), although all scores were within the normal range (score < 17).

### 3.2 Sleep results

The sleep characteristics in the nightmare group did not differ significantly to the ones observed in healthy controls (Table 1).

**Table 1:** Sleep characteristics in the 2 groups. The percentages of wake and sleep stages are in relation to the total bed time (TBT) of the two groups. Sleep efficiency (%) = Total sleep time/Total bed time for each participant ×100.

	Nightmare patients	Controls	Independent samples test
Total bed time (min)	513.8 ± 59.3	516.50 ± 53.6	*p* = .91 (*t* = −0.10)
Total sleep time (min)	431.7 ± 41.8	446.6 ± 48.5	*p* = .41 (*t* = −0.83)
Sleep efficiency (%)	84.1 ± 7.8	86.8 ± 8.5	*p* = .44 (*t* = −0.07)
Sleep latency (min)	10.4 ± 14.2	10.0 ± 8.0	*p* = .93 (*t* = 0.08)
Number of Awakenings	37.0 ± 14.0	30.8 ± 6.7	*p* = .20 (*t* = 1.3)
W%	12.0 ± 5.6	11.5 ± 8.1	*p* = .87 (*t* = 0.16)
N1%	7.1 ± 3.0	5.8 ± 1.9	*p* = .27 (*t* = 1.1)
N2%	44.6 ± 4.7	42.1 ± 7.1	*p* = .34 (*t* = 0.96)
N3%	16.2 ± 3.3	19.2 ± 4.6	*p* = .09 (*t* = −1.7)
REM%	20.1 ± 3.5	21.2 ± 6.3	*p* = .62 (*t* = −0.50)

### 3.3 Between–group HEP differences

To test the hypothesis that nightmare disorder might be characterized by increased neural responses to heartbeats, we contrasted the amplitude of HEP between nightmare and controls groups. The amplitude of HEPs significantly differed between the Nightmare and Control group during REM sleep (cluster-level *p* = .032, corrected for multiple comparisons in space and time). This effect was found over right-frontal regions (Fp2, Fz, F4, F8) and during the 449–504 ms post R-peak period (Fig. 1). No significant HEP differences were found in wakefulness or NREM sleep between both groups (both cluster-level *p*s > 0.5).

**Fig. 2:**
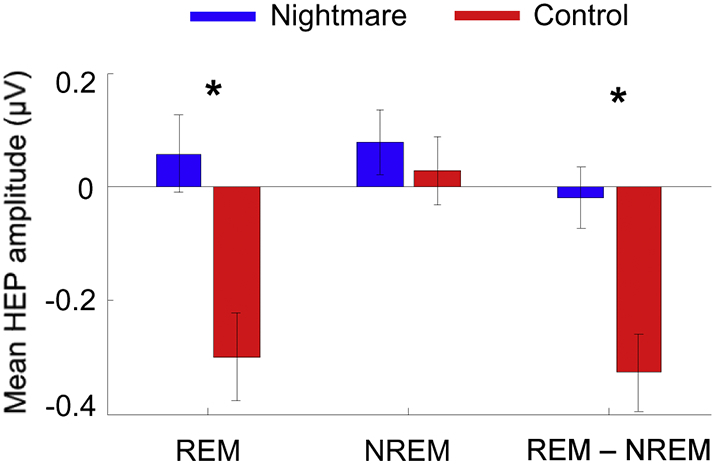
Mean HEP amplitude within the observed cluster during REM sleep, NREM sleep, and their difference for nightmare and control groups. A significant HEP difference between groups was observed during REM (two sample *t*-test, t_20_ = 3.46, *p* = .0025), but not NREM sleep (t_20_ = 0.61, *p* = .54). REM minus NREM HEPs differed between nightmare and control groups (t_20_ = 3.52, *p* = .0021).

We further tested whether the observed HEP difference within the cluster was specific for REM, but not for NREM sleep. For that, we first computed the mean HEP amplitude within the observed significant cluster for each of the four conditions (i.e., nightmare-REM, nightmare-NREM, control-REM, control-NREM). As shown in Fig. 2, a significant mean HEP difference between nightmare and control groups was observed during REM (two sample *t*-test, t_20_ = 3.46, *p* = .0025), but not during NREM sleep (t_20_ = 0.61, *p* = .54). In addition, difference of the mean HEPs between REM and NREM (i.e., REM – NREM) differed between nightmare and control groups (t_20_ = 3.52, *p* = .0021), further suggesting that the observed HEP difference between group was specific for REM sleep.

Next, we checked whether the observed HEP effect was driven by three nightmare patients who had nightmare in the laboratory. We therefore computed the mean HEP amplitude within the observed significant cluster, then compared them using a two sample *t*-test. Excluding these three patients, we still observed the differential HEP effect between the groups (two sample t-test, t_17_ = 2.70, *p* = .015).

We then ensured that the observed HEP effects were specifically associated with neural heartbeat signals, and did not merely reflect some persistent difference in neural activity between nightmare and control groups. We computed surrogate R-peaks that have same interbeat intervals as real R-peaks but were randomly shifted in time (−500 ~ +500 ms), and conducted the same HEP analysis repeatedly (100 times) (Park et al., 2016; Park et al., 2014). We found only one summed cluster *t*-statistics greater than the one computed from the real R-peaks, supporting that the observed differential HEP amplitudes are time-locked to the heartbeat (Monte-Carlo *p* = .0198).

**Fig. 3:**
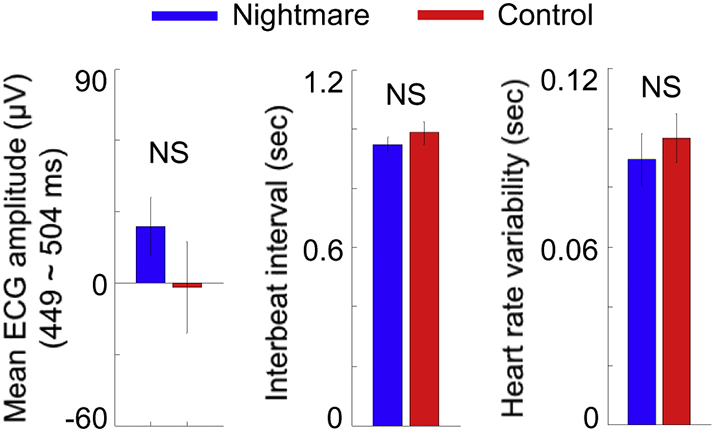
Other measured cardiac parameters did not differ between nightmare and control groups during REM sleep. ECG amplitude averaged over the time window where significant HEP difference was observed (left), interbeat interval (middle), heart rate variability (right). Error bars indicate SEM. NS, not significant.

Next, we verified that the differential HEP amplitudes between nightmare and control groups were not associated with differences in several basic cardiac parameters such as the ECG amplitude, interbeat interval, and heart rate variability (i.e., SDNN: standard deviation of normal-to-normal interbeat intervals). There were no such effects. The mean ECG amplitudes within the time window where HEP effects were found (449–504 ms post ECG R-peak) did not differ between nightmare and control groups (two sample *t*-test; t_20_ = 1.12, *p* = .28; Fig. 3), confirming that observed HEP difference did not reflect mere ECG artifacts. Moreover, neither interbeat interval (t_20_ = 0.82, *p* = .42; Fig. 3) nor heart rate variability (t_20_ = 0.61, *p* = .55; Fig. 3) during REM sleep differed between nightmare and controls.

### 3.4 Association between depression level and HEP amplitude in the frontal cluster

**Fig. 4:**
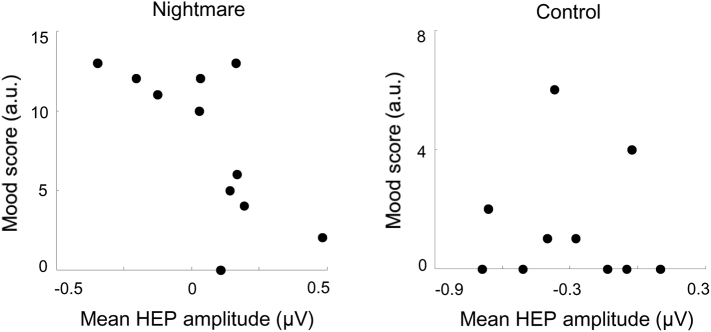
Mood score as a function of the mean HEP amplitude in the frontal cluster. Across participants, BDI score negatively correlated with the mean HEP amplitude within the significant cluster in the nightmare group (left, τ = −0.50, *p* = .03), but not in the control group (right, τ = −0.05, *p* = .84). Each dot indicates a subject.

Because of the non-normal distribution of the BDI scores in controls (Shapiro-Wilk normality test *P* < .05), the non-parametric Kendall's tau correlation coefficient was calculated between the BDI scores and the HEP scores in the frontal clusters for the 2 groups. Across the 11 nightmare patients, the mean HEP amplitude in the observed frontal cluster negatively correlated with BDI scores (τ = −0.50, *p* = .03; Fig. 4). No such correlation was observed in controls (τ = −0.05, *p* = .84; Fig. 4). In order to test if the correlation coefficients significantly differed, we first performed a conversion of tau values to Pearson r values (Walker, 2003), which yielded r_co_ = 0.08 and r_ni_ = 0.71, for the controls and nightmare patients, respectively. Then we used a Fisher r-to-z transformation for these r values, and the one-tailed *p* value for the difference between these coefficients was 0.059, which indicates a statistical trend for significance.

## 4 Discussion

The current study is, to our knowledge, the first to quantify neural responses to cardiac signals in patients with nightmare disorder and compare it with healthy controls. Our main finding is that patients with nightmare disorder demonstrate a stronger HEP response than healthy subjects in a frontal cluster, at a specific latency (449–504 ms post ECG R-peak) and only during REM sleep. As more positive HEP has been linked to heightened emotional arousal (Luft and Bhattacharya, 2015), motivation (Weitkunat and Schandry, 1990) and interoceptive awareness (Pollatos et al., 2005), this result supports that HEP may be used as a biomarker of increased emotional/reward and sensory processing during REM sleep in nightmare patients, and is in accordance with the observation that sensory processing sensitivity (Carr and Nielsen, 2017), including reward sensitivity (Perogamvros et al., 2015; Perogamvros and Schwartz, 2012), is amplified in nightmare patients. Higher HEP amplitude in a frontal cluster is consistent with a stronger engagement of frontal limbic cortical structures (e.g. anterior cingulate cortex) implicated in emotional/reward processing and negative emotions (Etkin et al., 2011) during the period of sleep associated with nightmares. Finally, we also found a negative correlation between frontal HEP amplitude and mood scores in the nightmare group as predicted, although the difference of correlations between groups showed only a statistical trend for significance.

### 4.1 Increased heartbeat-evoked potential amplitude during REM sleep in nightmare disorder

Hyperactivity of fear-related structures such as amygdala and anterior cingulate cortex (Braun et al., 1997; Maquet et al., 1996) and increased cortical excitability (Massimini et al., 2010) in REM sleep compared to NREM sleep and wakefulness are in line with the idea that REM sleep may offer a favorable neural condition for experiencing negative emotions in dreams and nightmares. Here, higher HEP in nightmare patients was restricted to REM sleep (and not NREM sleep or wakefulness), which supports the notion that nightmares are a typical REM parasomnia (American Academy of Sleep Medicine, 2014). However, note that in some cases (e.g. PTSD), nightmares were found to occur not exclusively during REM sleep but also NREM sleep (Woodward et al., 2000), and abnormalities in N2 sleep micro-structure (i.e. sleep spindles) have been reported in frequent nightmare recallers (Picard-Deland et al., 2018). In our study, increased HEP amplitude in REM sleep was found in nightmare patients, even after excluding from the analysis the patients who had a nightmare in the laboratory. This observation strongly supports the idea that the HEP effect was not primarily driven by the intense emotional arousal associated with the nightmares experienced during the experimental night by the excluded patients. Instead, our findings for HEP measures likely describe a (trait-like) physiological characteristic of REM sleep in nightmare patients, and not specifically periods with nightmares.

Depending on the cognitive or emotional state investigated, HEP modulations may engage several cortical structures, such as the posterior insula (Craig, 2002; Park et al., 2018), anterior cingulate cortex/ventromedial prefrontal cortex (ACC/vmPFC) (Park et al., 2014), amygdala (Mayer, 2011), somatosensory cortex (Kern et al., 2013) and the parietal cortex (Luft and Bhattacharya, 2015; Park et al., 2016; Park et al., 2014). For example, the frontal HEP component has been observed when people performed cardiac perception (Pollatos et al., 2005) and reward (Weitkunat and Schandry, 1990) tasks, while the parieto-occipital HEP component was found in visual perception tasks (Dirlich et al., 1998). Considering the limited spatial localization capacity of the current study and based on evidence from previous HEP work, we would like to cautiously suggest that the frontal cluster may result from ACC/vmPFC activity. Increased HEP amplitude in these regions has been associated with subjective experience and awareness (Park et al., 2014) and its presence in nightmare disorder is not surprising, as these structures are known to contribute to the appraisal and expression of negative emotion (Bissiere et al., 2008; Drevets et al., 2008; Etkin et al., 2011; Lindquist et al., 2012; Moser et al., 2015). The present HEP findings may thus reflect an increased salience assigned to incoming negative stimuli during aversive or threatening situations dreamed by the patients (Perogamvros and Schwartz, 2012). Indeed, HEPs are increased when levels of emotional arousal (Luft and Bhattacharya, 2015), motivation (Weitkunat and Schandry, 1990), or stress (Schulz et al., 2013) are high, as tested during wakefulness. Altogether, these findings support higher sympathetic nervous system reactivity and intensified emotional arousal in nightmare disorder. Importantly, heightened emotional reactivity has been also associated with adaptive functioning (Vonderlin et al., 2008), such as increased processing and awareness of the environment and enabling fast response to a threat, supporting the idea that nightmares may realize a biologically adaptive function, that of a threat simulation and preparation for future relevance (Perogamvros and Schwartz, 2012; Revonsuo, 2000) (see also below).

The role of frontal regions like the ACC in shaping the emotional arousal state in nightmares has been hypothesized in a previously proposed neurocognitive model of nightmares (Nielsen and Levin, 2007). In this model, a hyperactive ACC would augment *affect distress, which is a* trait-like factor consisting of a long-standing tendency to experience heightened distress and negative affect in response to emotional stimuli (Nielsen and Levin, 2007). *Affect* distress would thus determine the level of distress that one individual may experience both during and after a nightmare. Consistent with this hypothesis, as well as with our findings of increased HEP amplitude in frontal regions and with the prevalence of negative emotions in nightmares, the ACC is typically more activated in REM sleep than in wakefulness (Braun et al., 1997; Maquet et al., 1996). In addition, higher theta activity in a frontal region which roughly corresponds to ACC was found in REM sleep of nightmare patients compared to controls (Marquis et al., 2017).

Our finding may also suggest that, compared to control subjects, nightmare patients show increased responsiveness to sensory (including somatosensory or interoceptive) signals during REM sleep. Indeed, these results are in line with recent research claiming that *sensory processing sensitivity* is a trait marker that underlies the unique symptoms and imaginative richness found in individuals with nightmares (Carr and Nielsen, 2017). According to Aron et al. (Aron et al., 2012), sensory processing sensitivity is determined by four main factors: 1) stronger emotional reactions; 2) deeper cognitive processing of information; 3) greater awareness of environmental subtleties; and 4) becoming overwhelmed when stimuli are too strong. Increased sensory processing of both external stimuli and interoceptive signals may reflect enhanced cortical excitability and deficient inhibition of these signals (or increased attentional bias towards them), as found in a ‘classic’ disorder characterized by cortical and physiological hyperarousal, such as insomnia disorder (Colombo et al., 2016; van der Werf et al., 2010; Wei et al., 2016). Our results support that HEP can be used as a biomarker of increased emotional/sensory processing during REM sleep in nightmare patients and future research studying a potential HEP modulation with treatment for nightmares (e.g. prazosin or Imagery Rehearsal Therapy) (Aurora et al., 2010) would support this finding.

### 4.2 Impaired emotion regulation during dreaming in nightmare disorder

We also found that the more positive is the HEP amplitude in the frontal cluster in REM sleep, the less depressed were the nightmare sufferers (Fig. 4). Although this finding may have implications concerning the links between affective state in wakefulness and emotional arousal in REM sleep or dreaming (Perogamvros and Schwartz, 2012), we cannot draw any strong conclusion in this regard because the difference of correlations between groups showed only a statistical trend for significance. Therefore, it remains unclear whether nightmares, compared to normal dreaming, can offer an adaptive function (Revonsuo, 2000), similarly to normal dreaming (Perogamvros and Schwartz, 2012). It seems that such a role would diminish as nightmares become more severe or recurrent in nature. Indeed, although patients with idiopathic nightmares are normothymic, having nightmares is associated with an increased risk of developing PTSD upon subsequent trauma exposure (Mellman et al., 1995a) and nightmares following trauma are associated with more severe PTSD (Mellman et al., 1995b). These observations support that extinction learning fails when fear is exaggerated, as in nightmare sufferers. Furthermore, compared to controls, nightmare patients show decreased activity in regions associated with extinction learning (e.g. medial prefrontal cortex) at wake (Marquis et al., 2016) and impaired frontal inhibitory functions (Carr et al., 2018; Simor et al., 2012).

### 4.3 Limitations

The two main limitations of this study concern the low spatial resolution (20 electrodes) and the relatively small sample (11 patients, 11 healthy subjects). Regarding the precise localization of the frontal HEP, our hypothesis that the effect may be maximal in ACC/vmPFC should be taken with caution, although the frontal location of HEP was expected and concerned several frontal electrodes. The use of high-density EEG (>64 channels), as in other studies (Park et al., 2016; Wei et al., 2016), would increase spatial resolution and localization accuracy. On the other hand, while the sample size of this study was relatively small, also due to a very careful clinical selection of idiopathic nightmare sufferers, it is compensated by the very high number of intra-subject HEP measurements. We can therefore consider that the HEP results are robust.

### 4.4 Conclusions

In conclusion, the current findings support an increased interoceptive sensitivity in nightmare disorder, as indexed by the increased positive amplitude of a frontal HEP cluster in REM sleep. These results are in line with the idea that increased emotional arousal and sensory processing sensitivity participate in the pathophysiology of this sleep disorder and that the HEP may be a biomarker of this change.

## Funding

This work was supported by the Bertarelli Foundation, the Pictet Foundation, the BIAL Foundation grant (No 225/12) and the Swiss National Science Foundation (grant numbers: 320030_182497, 51NF40-104897, 320030-159862 and CR3113_149731).

## Declarations of interest

None.
